# Nanocrystalline cerium dioxide efficacy for gastrointestinal motility: potential for prokinetic treatment and prevention in elderly

**DOI:** 10.1186/s13167-015-0029-z

**Published:** 2015-03-11

**Authors:** Olena Yu Yefimenko, Yuliya O Savchenko, Tetyana M Falalyeyeva, Tetyana V Beregova, Nadiya M Zholobak, Mykola Ya Spivak, Oleksandr B Shcherbakov, Rostyslav V Bubnov

**Affiliations:** Taras Shevchenko National University of Kyiv, Volodymyrska Str., 64/13, 01601 Kyiv, Ukraine; Zabolotny Institute of Microbiology and Virology, National Academy of Sciences of Ukraine, Zabolotny Str., 154, 03680 Kyiv, Ukraine; LCL ‘DIAPROF’, Svitlycky Str., 35, 04123 Kyiv, Ukraine; Clinical Hospital ‘Pheophania’ of State Affairs Department, Zabolotny Str., 21, 03680 Kyiv, Ukraine

**Keywords:** Predictive, Preventive, Personalized medicine, Nanocrystalline cerium dioxide, Prokinetic, Smooth muscle, Stomach, Colon, Gastrointestinal motility, Rat model, Gastrointestinal cancer prevention, CRC prevention

## Abstract

**Background:**

Constipation is a common condition, with prevalence after 65 years, is a major colorectal cancer risk factor. Recent works have demonstrated advances in personalized, preventive nanomedicine, leading to the construction of new materials and nanodrugs, in particular, nanocrystalline cerium dioxide (NCD), having strong antioxidative prebiotic effect.

*The aim of our study* was to investigate the influence of NCD on motor function of the stomach and colon *in vivo* and contractive activity of smooth muscles in different year-old rats.

**Methods:**

We included 80 rats: 3- (weight 130–160 g, *n* = 40) and 24-month old (weight 390–450 g, *n* = 40), divided into four groups as follows: І—control group; rats of II–ІV groups were injected intragastrically one injection per day during 10 days, 3 ml of water 3 ml/kg stabilizing solution, аnd 1 mmol/ml NCD, respectively. In all animals, we recorded spontaneous and carbachol-stimulated (0.01 mg/kg) gastrointestinal tract motor activity. We used the index of motor activity (IMA), expressed in cmH_2_O, for characterization of the motor function. We investigated smooth muscle contraction by tenzometric method, studied the spontaneous and stimulated motility by ballonographic method.

**Results:**

IMA reduced by 21.1 + 0.2% (*p* < 0.01) in the old rats of the control group compared with the young rats. A 10-day administration of NCD increased IMA in the stomach of young rats by 9.3% (*р* < 0.001) vs the control group. The exposure of NCD increased the amplitude of contraction to 34.2 ± 5.4 mN (*n* = 10) in the stomach of old rats and increased by 32.1 ± 2.4% vs the control group (*p* < 0.05). NCD did not influence acetylcholine (ACh) contractions in the stomach of young rats; however, in the stomach of old rats, *V*_nr_ increased by 90 ± 15.2% (*р* < 0.001).

**Conclusions:**

The index of motor activity is decreased in old rats. Nanocrystalline cerium dioxide increased the index of motor activity in all groups of rats and also evoked a significant increase of colon contractions in old rats.

## Overview

### Predictive, preventive and personalized medicine in gastrointestinal motility and gastrointestinal cancer prevention

In different countries, every third or fourth adult regularly or occasionally suffers from constipation [1]. Moreover, there is a sharp increase in their frequency after 65 years [2]. It is known that constipation is a major risk factor for colorectal cancer, because of the increased level of carcinogenic metabolites in the colon and increased time of contact metabolites with intestinal mucosa [3].

The role of predictive, preventive and personalized medicine for the human population to live longer, and healthcare systems around the world is challenged to find new ways to improve global health, dealing in particular with gastrointestinal cancer, colorectal cancer (CRC) [4,5]. Therefore, it is imperative that scientists and clinicians collaborate closely in new developments in a wide scope of complicated underlying mechanisms of carcinogenesis in the vision of integrated medicine.

An actual problem of modern biomedicine is the development of new prokinetics, such as majority of novel drugs which have significant side effects [6,7]. Analysis of existing motility promoters shows that the prebiotics have least side effects. It is known that prebiotics activate motility and evacuation of the colon through the formation of short-chain fatty acids [8].

Advances in nanoscience, nanotechnology and nanomedicine lead to the construction of new materials and devices for various scientific and therapeutic purposes which are applicable in molecular diagnostics, nanodiagnostics and improvements in the discovery, design and delivery of drugs, including nanopharmaceuticals.

Recent works have demonstrated that nanocrystalline cerium dioxide (NCD) has prebiotic effect; however, its prokinetic properties still were not studied.

Thus, the *aim of our study* was to investigate the influence of NCD on motor function of the stomach and colon *in vivo* and contractive activity of smooth muscles in different year-old rats.

## Methods

The investigations were carried out on 80 rats maintained in accordance with guidelines of Animal Ethical Research Committee of Taras Shevchenko National University of Kyiv. Protocol of Ethics Committee was N8 from 03.04.2014. Animals were divided into four groups: 3- (weight 130–160 g, *n* = 40) and 24-month old (weight 390–450 g, *n* = 40). All rats were deprived as follows: І—control group; ІІ—group which were injected 3 ml of water (intragastrically, one injection per day); ІІІ—rats which were injected 3 ml/kg stabilizing solution (intragastrically, one injection per day); аnd ІV—group of rats which were injected with NCD in a dose 1 mmol/ml (intragastrically, one injection per day). All drugs were administrating during 10 days.

Rats maintained on hunger with free access to water for 12 h. Motor activity of the stomach and colon was registered by ballonographic method in rats under urethane anesthesia (Sigma, USA) (1.1 g/kg, intraperitoneally (i/p) [9,10]. Tracheotomy was performed. An intragastric and colon balloon created from thin latex rubber connected with plastic tubing was introduced into the stomach and colon. In young animals, the balloons were filled with 1 and 0.5 ml warm water (37°C) accordingly. In old rats—1.5 and 0.7 ml, respectively. This volume range was determined to be the level required to induce an intragastric and intracolon pressure of 10–11 cmH_2_O. Spontaneous motor activity during 120 min of the animals of all groups after the 20-min period was recorded. Afterwards, it was injected with standard stimulant of motility carbachol (0.01 mg/kg, i/p, Sigma, USA) and carried out further record. We used the index of motor activity (IMA) for characterization of the motor function of the gastrointestinal tract. IMA was expressed in cmH_2_O.

The investigation of smooth muscle contraction of the stomach and colon was performed according to the method described in [11]. In the experiment, we used circular smooth muscle strips (medium size—2 × 10 mm), cleaned of mucosa, which were placed in a chamber with flowing Krebs solution of 5 ml (flow rate—5 ml/min). Strips gave passive tension (10 mN) and left for 1 h. The contractile activity was studied in isometric mode using force sensors. Signals were recorded using an electric potentiometer N339 (Russia). We selected classic stimulants: hyperpotassium isotonic solution (HPS) and acetylcholine (ACh). Analysis of contractile responses of smooth muscle preparations were carried out according to the method described in the article [11]. This method is based on the transformation phase reduction and relaxation that are S-shaped curves and can be mathematically described by the general formula:

$$ f={f}_m\frac{\tau^n}{\tau^n+{t}^n} $$

where *f* is the instantaneous (at time *t*) force, *f*_m_ is the maximum force, *τ* is the characteristic time (numerically equal to the time at which there is half the maximum value of the force ½ *f*m) and *n* is the logarithmic coefficient slope of mechanical kinetics curve.

The method involves linearization of phase relaxation of mechanical kinetic curve in the coordinates {ln[(*f*m − *f*)/*f*]; ln *t*}, where *f*_m_ is the value of the maximum power of contraction. Time, which is achieved *f*_m_, taken initial phase relaxation reference point *t* = 0; current value of time *t* corresponds to the value of the instantaneous force *f*. From the linearized curve, we determined kinetic parameters: ln is the segment that cuts on the *x*-axis line drawn through the experimental points and an empirical parameter *n* is the slope of the line that describes the steepness dependence *f* (*t*). Kinetic value meaning of the characteristic time of relaxation phase *τ*, that is, the time during which the mechanical muscle tension decreases to a level ½*f*_m_. The main kinetic characteristic of the process of relaxation is maximum speed of the process. This method allows to calculate the amplitude-independent index-normalized maximum relaxation rate.

We used the following indicators of contractile activity of smooth muscle preparations cuts: amplitude, ratio phase and tonic components, phase contraction (*V*_nc_) and relaxation (*V*_nr_) of maximal normality velocity.

NCD synthesized by methodology [12] in Zabolotny Institute of Microbiology and Virology [13].

Our date were normally distributed by test Shapiro-Wilks’ *W*-test. All results are expressed as the M ± SD of *n* values. Statistical comparisons between groups were conducted using Student’s *t*-test for unpaired data. Statistical significance was set at *р* < 0.05 [14].

## Results

**Figure 1:**
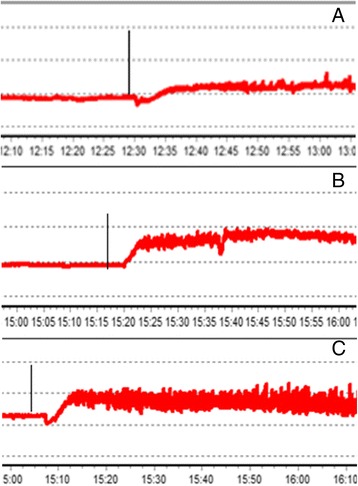
**A typical record of motor activity of the stomach in rats. (A)** Three-month rat; **(B)** 24-month rat; **(C)** 24-month rat after 10-day administration of nanocrystalline cerium dioxide; introduction of carbachol at a dose of 0.01 mg/kg, ip.

*In vivo* studies of spontaneous gastric motility have shown that IMA was reduced by 21.1 ± 0.2% (*p* < 0.01) in old rats of the control group compared with the young rats. A 10-day administration of NCD increased IMA in the stomach of young rats by 9.3% (*р* < 0.001) relatively to the control group (Figure 1).

**Figure 2:**
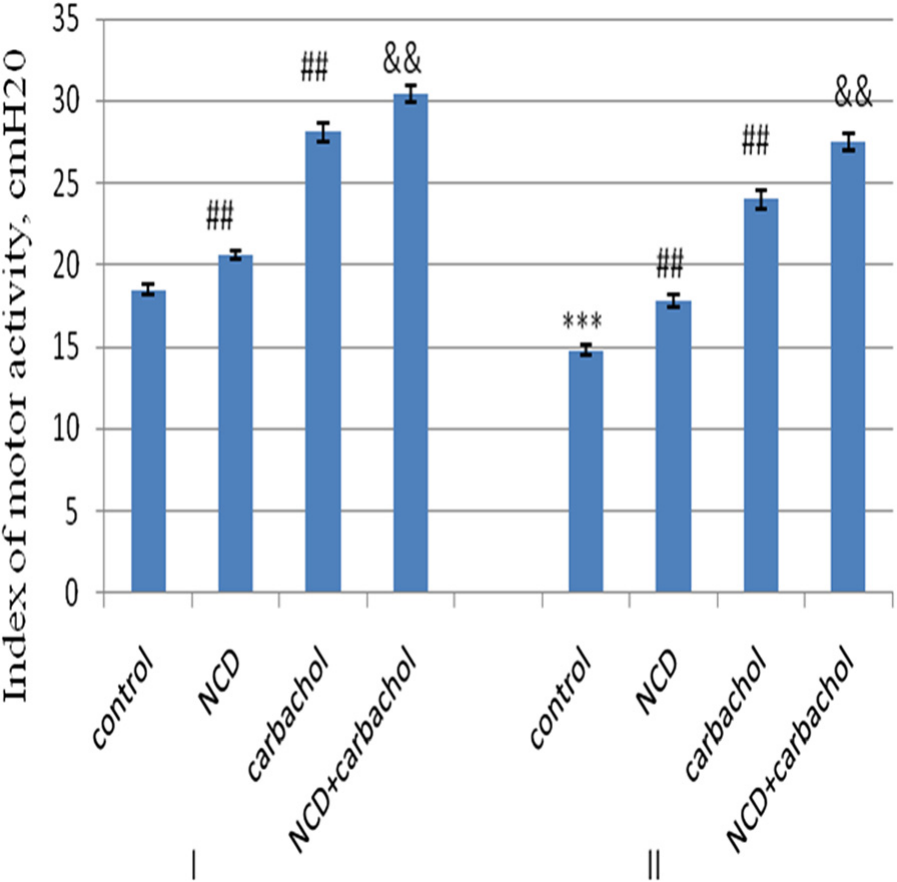
**Index of motor activity of colon 3-month-old (І) and 24-month-old rats (ІІ).** M + SD, *n* = 10, ***р < 0.001, 24-month-old rats compared with 3-month-old rats; ^##^ *р* < 0.01, compared with the corresponding control groups of rats, &&*p* < 0.01, respectively, to carbachol.

Introduction of NCD also increased the motor activity of the stomach of old rats: IMA increased by 19.8 ± 0.3% (*p* < 0.001). Thus, the effect of strengthening of IMA by NCD was almost twice higher in the old rats than in the young. In old rats, NCD almost restored the value of IMA targets of young animals (Figure 2).

The introduction of carbachol to young rats caused a marked motor response in the stomach. IMA under carbachol action was driven by 34.2 ± 0.4% more than in terms of spontaneous motility. IMA was lower by 14.6 ± 0.1% (*p* < 0.01) in response to carbachol in the old rats than in the young. IMA increased after the 10-day administration of NCD in rats of both age groups. In young rats, IMA increased by 8.2 ± 0.08% (*p* < 0.05) in old—14.5 ± 0.2% (*p* < 0.05) (Figure 2). Thus, NCD amplified the effect of carbachol in young and old rats.

**Figure 3:**
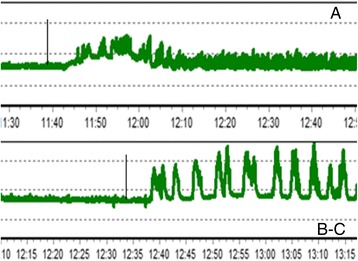
**A typical record of motor activity of the colon in rats. (A)** 3-month rat; (**B)** 24-month rat; **(C)** 24-month rat after 10-day administration of nanocrystalline cerium dioxide; introduction of carbachol at a dose of 0.01 mg/kg, ip.

In rats 24 months of age, IMA was 14.1 ± 0.3% (*p* < 0.001) lower in the colon relative to the control group of young rats. The 10-day introduction of NCD increased IMA by 13.4 ± 0.09% (*p* < 0.001) and 14.3 ± 0.1% (*p* < 0.001) in young and old rats, respectively, compared with the corresponding control groups (Figure 3).

**Figure 4:**
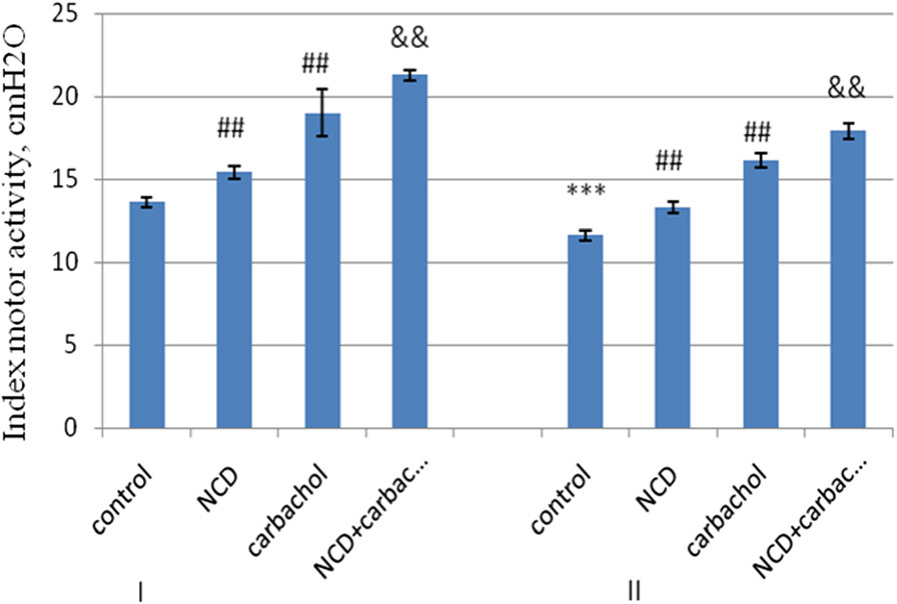
**Index of motor activity of colon 3-month-old (І) and 24-month-old rats (ІІ).** M + SD, *n* = 10, ***р < 0.001, 24-month-old rats compared with 3-months of age rats; ^##^ *р* < 0.01, compared with the corresponding control groups of rats; &&*p* < 0.01, respectively, to carbachol.

Motor function of the digestive tract decreases with age. In the old rats, the response to carbachol was also weaker than in the young. NCD affect both basal and stimulated motility in the stomach and colon. NCD restored the indicators of motor activity in old rats to targets of young animals (Figure 4).

The studies on gastric smooth muscle preparations showed that HPS-caused contraction characterized by an average amplitude was 18.6 ± 11.7 mN, and the ratio of phase and tonic components was 3.2 ± 3.5% (*n* = 10). Aging did not cause changes in these parameters. Functional activity of HPS-induced contractions of smooth muscles of the stomach in a group of old rats underwent no significant changes compared with those of young animals, but tended to decrease (Table 1).

**Table 1:** **Indicators of HPS-induced contractions of stomach smooth muscles in rats (n = 10)**

**Parameter**	**3-month-old rats**	**24-month-old rats**
Amplitude (mN)	18.6 ± 7.8	25.9 ± 4.8
Tonic/phase components (%)	3.2 ± 3.5	4.5 ± 3.5
Velocity of contraction (*V*_nс_) (min^−1^)	2.1 ± 0.8	1.8 ± 0.2
Velocity of relaxation (*V*_nr_) (min^−1^)	0.74 ± 0.2	0.57 ± 0.1

The 10-day administration of NCD increased the ratio of phase and tonic components by 21.9 ± 1.1% (*p* < 0.05) in the stomach of young rats. All other parameters, NCD had no statistically significant effect.

**Figure 5:**
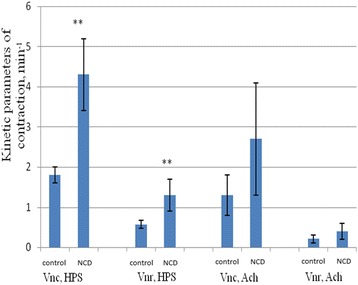
**The influence of nanocrystalline cerium dioxide (NCD) on phase of contraction and phase of relaxation of HPS and ACh-induced contractions.** M ± SD, *n* = 10, **р < 0.01 compared with the corresponding control groups.

The exposure of NCD increased the amplitude of contraction to 34.2 ± 5.4 mN (*n* = 10) in the stomach of old rats, which corresponded to an increase by 32.1 ± 2.4% compared with the control group (*p* < 0.05). The value of the phase and tonic components in old rats increased to 10.0 ± 3.8% after the actions of the NCD, which was more than 122 ± 7.9%, compared to young rats (*p* < 0.01). The kinetic parameters of HPS-induced contractions statistically significantly increased under action of NCD in the stomach of old rats. The rate of development of contractile responses (*V*_nc_) increased by 138.8 ± 11.2% (*p* < 0.01), and the rate of phase relaxation (*V*_nr_) by 128.1 ± 10.8% (*p* < 0.01) (Figure 5).

ACh-induced contractions had amplitude 9.6 ± 3.8 mN (*n* = 10) in young rats, but in old, it was 39.7 ± 4.1 mN (*n* = 10), which was higher by 313% (*р* < 0.001). The value of the phase and tonic components had a tendency to increase with age from 34.5 ± 12.8% to 49.4 ± 12.7% (*p* > 0.05) (n = 10). *V*_nс_ of АCh-induced contractions had no difference between the young and old rats in the stomach. But *V*_nr_ decreased with age by 57 ± 8.6% (*р* < 0.05) (Table 2).

**Table 2:** **Parameters of АCh-induced contractions of smooth muscles of stomach in rats (** ***n*** **= 10)**

**Parameter**	**3-month-old rats**	**24-month-old rats**
Amplitude (mN)	9.6 ± 3.8	39.7 ± 4.1**
Tonic/phase components (%)	34.5 ± 12.8	49.4 ± 12.7
Velocity of contraction (*V*_nс_) (min^−1^)	1.9 ± 0.7	1.3 ± 0.5
Velocity of relaxation (*V*_nr_) (min^−1^)	0.49 ± 0.3	0.21 ± 0.1*

*р < 0.05, **р < 0.001, compared with the control group of 3-month-old rats.

NCD did not influence ACh contractions in the stomach of young rats; however, in the stomach of old rats, *V*_nr_ increased by 90 ± 15.2% (*р* < 0.001) (Figure 3). All other parameters had no significance.

Contractile activity of HPS-stimulated contraction is caused by Ca^2+^ ions which enter the smooth muscle cells through potential-induced Ca^2+^-channel L-type [13]. We assume that the mechanism of effects of NCD in the stomach is mediated through the Ca^2+^ ions, due to all indicators of HPS-induced contractions that were growing after the 10-day administration of NCD in both age groups and were not the changes of Ax-induced contractions (except the phase of relaxation).

The investigation of parameters of motor activity of the colon showed that HPS-induced contraction of smooth muscles of young animals characterized by the average amplitude of 13.5 ± 5.4 mN (*n* = 10). Value of phase and tonic components of the contractions was 12.3 ± 8.2%. In the case of the colon, smooth muscle of old rats HPS-amplitude contractions remained close to young animals (10.8 ± 5.1 mN, *n* = 10), and the ratio of phase and tonic components tended to decrease (7.9 ± 4.1%, *n* = 10, *p* > 0.05).

**Figure 6:**
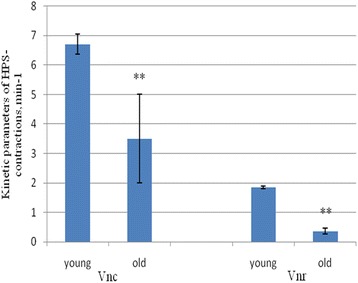
**The changes of phase of contraction and phase of relaxation of HPS-induced contractions with age.** M ± SD, *n* = 10, **р < 0.01.

The applied kinetic analysis showed that with age, the phase of reduction decreased by 47.8 ± 7.4% (*p* < 0.01), and phase relaxation by 81.1 ± 13.2% (*p* < 0.01) (Figure 6).

These data indicate disrupted Ca^2+^ entry to the smooth muscle cells through Ca^2+^-channel L-type in the colon of rats with age. The proof of our assumptions is literature data, which found that with reduced age, the level of intracellular calcium in smooth muscle cells of the colon [15] and acetylcholine release reduced by 50% in electrically stimulated preparations of the colon [16]. In addition, researchers found that K^+^ and Ca^2+^ currents through appropriate channels reduced in the smooth muscle cells of the colon of old rats that influence the onset reduction [17].

**Figure 7:**
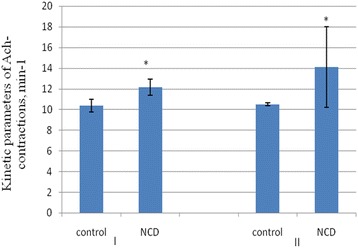
**The influence of NCD on phase of contraction and phase of relaxation of HPS-induced contractions in old rats.** M ± SD, *n* = 10, **р < 0.01.

NCD administration during 10 days did not cause the changes in indicators of HPS-induced contractions of smooth muscle of the colon in young rats. Whilst in the old rats, the amplitude increased from 11.5 to 37.9 mN (*n* = 10), which was more than 350% (*p* < 0.001). The value of phase and tonic components of HPS-activated contraction was 12.58 ± 4.95% (*n* = 10), which was more by 59.5 ± 9.1% (*p* < 0.05) in the case of control animals. In the group of old rats, *V*_nc_ and *V*_nr_ increased by 166.8 ± 21.4% and 644 ± 33.6%, respectively (*p* < 0.001) (Figure 7).

In the control group, an amplitude averaged 8.7 ± 2.2 mN (*n* = 10), and the ratio of phase and tonic components were 24.6 ± 8.9% (*n* = 10) of ACh-induced contractions of smooth muscle of the colon in young rats. With age, the amplitude of ACh smooth muscle contractions of the colon increased to 15.6 ± 10.2 mN (*n* = 10), but the ratio of phase and tonic components of the contractile response decreased to 10.9 ± 0.7% (*n* = 10). These data coincide with studies where it was shown that ACh increased the strength of contractions in the colon of young and old rats [18]. In old rats, kinetic characteristics of ACh contractions remained similar to the data obtained from the intestine of young animals.

The 10-day NCD administration caused the changes of the parameter of ACh-induced contractions. In young rats, the amplitude of ACh contractions increased by 177 ± 23.6% (*p* < 0.001). The value of the phase and tonic components of the contractile responses also increased by 30.9 ± 6.8% (*p* < 0.05). In old rats, these values tended to increase. Namely, the amplitude Ax-induced contractions increased from 15.5 ± 10.2 to 16.9 ± 4.3 mN (*n* = 10) (*p* > 0.05), and the ratio of phase and tonic components by 79 ± 7.6% (*p* < 0.05). Since the phase ratio and tonic components ACh contractions is an indicator of the efficiency of the activation signal via muscarinic acetylcholine receptors [19], our results suggest efficiency improvements activation of the signal after exposure of the NCD.

**Figure 8:**
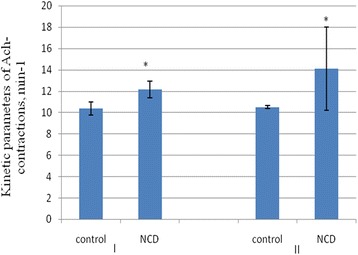
**The influence of NCD on phase of contraction of ACh-induced contractions in young (I) and old (II) rats.** M ± SD, *n* = 10, *р < 0.05.

Kinetic parameters also undergo changes. Speed of the contraction increased by 17 ± 4.3% and 34.3 ± 3.6% (*n* = 10), respectively, after administration of NCD in young and in old rats (Figure 8). However, no statistically significant changes occur in the phase of relaxation of ACh-induced contractions under action NDC.

## Discussion

Thus, NCD increased IMA of spontaneous and stimulated motility in rats of both age groups *in vivo*. In smooth muscle preparations, NCD affect parameters of HPS-induced contractions in both age groups and the phase of relaxation of ACh-induced contractions in old rats. In view of this, we can say that the mechanism of NCD is caused by Ca^2+^ ions which enter to the smooth muscle cells through potential-induced Ca^2+^-channel L-type in the stomach.

In old rats, a significant strength of the contractions under action of NCD of the colon, activated by depolarization of the plasma membrane, was observed. Since Ca^2+^ ions that enter through potential-induced L-type myocyte Ca^2+^-channel make the main contribution to the generation of these cuts in the smooth muscles of the gastrointestinal tract, it can be assumed that they are one of the possible effector units under the action of NCD. It should be noted as a significant acceleration of the process of relaxation of hyperpotassium contraction in a group of old rats that received NDC. It can be associated with a number of cellular processes; in particular, this effect may indicate activation of Ca^2+^ pumping from myoplasma after excitement (possible effector proteins—Ca^2+^-pump plasma membrane and/or the sarcoplasmic reticulum and the Na^+^, Ca^2+^ exchanger). We cannot discard the effect of NCD through the activation signal of muscarinic acetylcholine receptors as NCD increased parameters of ACh contractions. Thus, NCD mediates its effects through multiple effector units in the colon.

NCD may potentially act through the muscle, neurons and amine precursor uptake and decarboxylation (APUD) cell. Antioxidative actions of nanoceria on molecular and subcellular level are the potential clues for understanding the mechanism of cancerogenesis.

For this reason, multiparameter study of colon motility gives a new insight to digestive cancer pathogenesis understanding, modeling and development of personalized treatment, and in the field of stress, visceral pain, vasospasm, physical activity and nutrition within the integrated concept of predictive, preventive and personalized medicine (PPPM).

### Nanotechnologies—the challenge for advanced diagnosis, treatment and prevention

Pharmacological, pharmaceutical and toxicological aspects of the application of nanoparticles in biomedical purposes still remain poorly understood. The application of nanoparticles allowing the combination of therapy and diagnosis, known as theranostic, has received increasing attention in biomedicine [12,13,20], whilst oxidative stress has been postulated as one of the main physiopathological hallmarks of most of chronic diseases.

The application of nanoceria under conditions of involving oxidative stress can reduce/remove its damaging effects, thus providing protection to the organism from adverse environmental factors: UV irradiation, viral, bacterial, fungal lesions induced toxic effects and pathological conditions associated with aging. Nanoparticles of cerium dioxide, considering its UV-shielding effect, antiviral, antibacterial, antifungal activity, cardioprotective, neurotrophic, hepato- and nephroprotective, and anti-aging effect, have potential for various biomedical applications [12,13,20]. Treatment with nanoceria has supplementary perspectives in gynecology and reproductive medicine and also in women with hormone-associated obesity which results from the increase in the number of oocytes in follicles, increase in the number of oocytes at metaphase I and metaphase II, increase in the number of living granulosa cells, and decrease in the number of necrotic and apoptotic cells [13]. Musculotropic action of gold nanoparticles [21,22] was reported as strong agents against oxidative damage having anti-aging activity. Cerium dioxide-based nanomaterials are also very promising in cancer theranostics. Due to their unique properties, CeO_2_ nanoparticles are already used as a means of drug delivery to the tumor site and as a diagnostic tool (either alone or as part of the existing compositions), and as a means of cancer treatment [12,13,20].

### Gut microbiota and motility, obesity, vasospasm and congestion in integrated PPPM vision for management of digestive disorders

The gut microbiota can be considered an extension of the self and, together with the genetic makeup, determines the physiology of an organism, metabolism and digestion [23]. Strachan [24] described the *hygiene hypothesis* that refers to an originally associated reduced microbial contact to microbes in early life and is suggested to be one of the main mechanisms to account for the increasing prevalence of allergic diseases over the past few decades. Today, reduced microbial exposures (and the rise in allergic conditions) have been attributed to Western lifestyle factors such as diet, antibiotic use, vaccinations, reduced household size and improved hygiene [24]. The probiotic bacteria have been widely suggested to affect obesity and metabolic disorders [25].

*Endothelial dysfunction* (ED) is an important risk factor that impairs blood flow controls in various organs. Obesity impairs microvascular function in several ways. ED results from an imbalance between nitric oxide (NO) and endothelin (EDN), being the regulators of vascular function. ED is associated with decreased NO production due to impaired endothelial NO synthase activity and expression, and increased production of superoxide anion and the endogenous NOS inhibitor ADMA, together with increased vasoconstrictor factors, such as endothelin-1 and sympathetic nerve activation [26].

Genetic variants in NO synthase and EDN isoforms and its receptors (EDNRA and EDNRB) appear to account for important components of the variance in ED, particularly when concurrent risk factors such as obesity exist. Analysis of genotype-phenotype interactions is critical for the formulation of the potentially altering predisposition to cardiovascular diseases [27]. NO synthase and endothelin genes are related with many diseases, namely, asthma [28] and renal failure [29], that make them the potential biomarkers of numeral obesity collateral pathologies.

Meyer et al. found that in the aorta of obese mice, perivascular adipose potentiates vascular contractility to serotonin and phenylephrine, indicating the activity of a factor generated by perivascular adipose, which was designated as ‘adipose-derived contracting factor’ (ADCF) [30]. Inhibition of cyclooxygenase (COX) fully prevented the ADCF-mediated contractions, whereas COX-1 or COX-2-selective inhibition was only partially effective. By contrast, inhibition of superoxide anions, NO synthase, or endothelin receptors had no effect on ADCF activity [31].

*Congestive mesenteric* [32] and/or *pelvic syndromes* (ovarian vein reflux) [31] are the condition characterized by the presence of venous congestion and varicose veins in the mesenteric and pelvic region, and play important role for dysregulation of intestinal and systemic microcirculation mechanisms leading to ED and have potential risk for the development of many vascular and hormonal disorders. In obese individuals, the mixed meal drink decreases the baseline skin perfusion and causes acetylcholine-mediated vasodilation, but has no effect on the capillary density. Obese individuals had impaired acetylcholine-mediated vasodilation after meal ingestion. The latter findings are consistent with impaired postprandial microvascular function in obesity [33,34].

Peripheral microcirculation assessment might be considered to support a supplementary information for obese patients particularly for vasospasm assessment [35], including laboratory biomarkers and capillaroscopy [36]; Doppler techniques for assessment of vascular responses following cuff-induced arterial occlusion allow determinations of the kinetics of post-ischemic reperfusion and provides an accurate reporter of NO-mediated physiological recruitment [27].

Pathways between skeletal muscle spasticity and with visceral, myofascial pain [36] and also with *Flammer syndrome* [37] might give interesting pathogenesis clues to understanding this psyche-intestine-circulation-pain interaction as a whole.

### CNS and enteric neuroregulation of digestive motility—insight for carcinogenesis—and challenge for PPPM

The digestive system is innervated through its connections with the central nervous system (CNS) and by the enteric nervous system (ENS) within the wall of the gastrointestinal tract [38]. The ENS is located within the wall of the gastrointestinal tract and responsible for its physiological functions such as secretion, blood flow and motility [39,40], working in concert with CNS reflex and command centres and with neural pathways that pass through sympathetic ganglia to control digestive function have a high affinity for the peripheral nervous system (PNS), may also react within the ENS, which is the largest and most complex subdivision of the PNS [38]. Damage to enteric neurons, or changes in their electrophysiological properties, results in altered and deficient physiological functions of the gut which has been demonstrated in studies of intestinal inflammation [41,42]. Deficient physiological functions have been demonstrated as a result of damage to neurons in the ENS of rats following long-term cisplatin administration [39,41].

Interaction of neural tissue motility and carcinogenesis was studied in several papers of Ceyhan et al. [43-46] and the ‘neural remodeling’ was described in carcinogenesis in the pancreas that implies that the local activity of the autonomic nervous system may be subject to an inhibition, which is why studies on the effect of (autonomic) neurotransmitters on cancer cells and in murine pancreatic cancer models which do not reflect pancreatic neuroplasticity should be interpreted and translated cautiously.

Authors assumed that:

Firstly, neurotransmitters can directly induce cell migration or regulate other parts of the metastasic multi-step process. Secondly, tumor cells can use nerve fibers as routes for invasion and emigration from the primary tumors*.*

The latter is of course experimentally difficult to handle, and there are only few methods established on this. One of the most advanced methods is probably that used by Ayala et al. who co-cultured dorsal root ganglia from mice with tumor cells [47,48].

Nerves in cancerogenesis were reported as a rich source of neurotrophic factors like nerve growth factor (NGF), glial-cell-derived neurotrophic factor (GDNF), artemin; of neuronal chemokines like fractalkine; and of autonomic neurotransmitters like norepinephrine which can all enhance the invasiveness of cancer cells via matrix-metalloproteinase (MMP) upregulation, trigger neural invasion (NI) and activate pro-survival signaling pathways [43].

These data of evidence supports a longstanding hypothesis that chronic stress can influence tumor growth and progression [44].

### Neuroendocrine, APUD cells signaling, serotonin, substance P

Neuroendocrine, APUD cells signaling and serotonin are important and not sufficiently studied mechanisms for number of pathologies of different localization, and link amongst series of pathological processes as obesity gut motility, cancer, etc. Serotonin is a primal signaling molecule conserved across phyla that is implicated in the control of energy balance [49-52]. Observations suggest that constitutive gastrointestinal motility depends more on neuronal than EC cell serotonin; moreover, serotonergic neurons promote development/survival of some classes of late-born enteric neurons, including dopaminergic neurons, which appear to innervate and activate in the adult ENS. As obesity increases peripheral serotonin, the inhibition of serotonin signaling or its synthesis in adipose tissue may be an effective treatment for obesity and its comorbidities [53]. Crane et al. [53] have found that genetic or chemical inhibition of Tph1 protects or reverses the development of HFD-induced obesity and dysglycemia via activation of UCP1-mediated thermogenesis. Thus, inhibiting Tph1-derived serotonin may be effective in reversing obesity and related clinical disorders such as NAFLD and type 2 diabetes.

Still interesting as a substrate for research and potential biomarker remains *substance P*, involved in nociception, and transmitting information about tissue damage from peripheral receptors to the central nervous system to be converted to the sensation of pain [54]. It has been theorized that it plays a part in fibromyalgia [55,56] and showed prokinetic properties, whilst was found to secrete serotonin as well as substance P and neurotensin by intestinal protozoa [57]. Substance P activates colonic motility via a direct action on colonic muscles over the whole colonic length and by simultaneous activation of neural cholinergic excitatory pathways in the middle and distal of noncholinergic excitatory pathways in the proximal colonic segment, and by activation of nitric oxide-dependent inhibitory neural pathways [58]. Substance P also has effects as a potent vasodilator, inducing vasodilatation is dependent on nitric oxide release [59].

### Potential advantages over existing laxatives, translation and implementation

Chronic idiopathic constipation, being a common functional disorder of the gastrointestinal tract, is a condition difficult to treat. Many of the novel treatment techniques in spite of showing their effectiveness still lack rigorous scientific support (level I evidence), and that means that at least randomized controlled trials have been performed and the treatment approach has been found to be effective. However, today, many of the usual every day practice treatments including using laxatives still are not supported by *level I evidence* [60-69].

Despite the widespread use of laxatives by health professionals to manage constipation, there has been a long standing lack of evidence to support this practice. There is little shared understanding between patients and professionals about ‘normal’ bowel function with little consensus in general practice of the optimum management strategies for chronic constipation and the most effective strategies to use. Chronic constipation still has not been an agreed management target within national frameworks [60]. Thus, in review by Gordon et al., polyethylene glycol preparations may increase the frequency of bowel motions in constipated children. Polyethylene glycol was generally safe, with lower rates of minor side effects compared to other agents. Common side effects included flatulence, abdominal pain, nausea, diarrhea and headache. Common *side effects* with liquid paraffin included abdominal pain, distention and watery stools.

There was no evidence to suggest that lactulose is superior to the other agents studied, although there were no trials comparing it to placebo [61]. Evidence for any benefit of laxatives is conflicting [62], and there has been no definitive summary of the evidence.

Thus, the use of laxatives still has a number of limitations and side effects and problem to cumulate evidence.

Patient preferences and the absence of patient equipoise formed an enormous barrier to the recruitment of patients in the implementation of the trial. Studies are needed to investigate different methods of recruitment within the constraints of current ethical guidelines on ‘opting in’ and to identify barriers and facilitators to recruitment to complex trials in general. Patient preference trials and natural cohort observational studies are also needed to investigate the effectiveness or cost-effectiveness of different laxatives and treatment strategies in the management of chronic constipation [61]. The majority of patient-recruited trials were in an institutional setting, such as a nursing home or hospital. Ten trials compared one laxative agent with another. The mean age of participants in these trials was estimated at 77 years. Only one trial examined patients in an outpatient setting; the other trials were carried out in nursing homes or hospitals [63]. In addition, these studies were relatively short in duration, and so it is difficult to assess the long-term effectiveness of these agents for the treatment of constipation. Long-term effectiveness assessment is essential, given the often chronic nature of this problem to prove effectiveness over placebo control for treatment to prevent one patient failing to respond to therapy [64].

Considering the results of current research and series of hypothetical issues, developed nanocrystalline cerium dioxide-based prokinetic agents should have potential advantages compared to the existing laxatives on the market being safe and biocompatible; have no (few—additional studies necessary) side effects; acts on several chains on pathogenesis; have harmonized multifactorial local and systemic antioxidative action; potential preventive effects; have prebiotic properties, may be used as food supplement with probiotics and sophisticated personalized diets programming; and are inexpensive*.*

Future research planning should focus on methods of translating obtained data to human organism and should initiate related research towards increasing level of evidence for promising and effective treatments that are still not sufficiently supported by evidence and adhere study protocols to the PPP medicine, including 1) *in vitro* studies; 2) *in vivo* studies in laboratory animals; 3) epidemiological studies; 4) studies on human volunteers and patients with relevant assessment of efficacy of each particular case.

High-quality *basic research* are needed (including molecular, immunohistochemical, preclinical imaging microbiology study); tests of safety and biocompatibility of the product with development and standardization of a protocol to perform predictive modeling of the GI function and prokinetic intervention; identification of the associations that are specific to the models and distinguish from those that are specific for humans; and identification of the associations that are common to humans and animal models with focus on mechanism in elderly, carcinogenesis in order.

*Clinical studies* on volunteers and recruited patients with sufficient methodological quality should include psychoemotinal and physical assessment and statistical issues that are associated with modifiable factors (nutrition, physical activity, behavior, etc.), with caution due to potential inaccuracies to avoid potential *limitations* inherent to dietary research, constipation studies (as intention-to-treat analysis, relevant long-term symptoms assessment, placebo control, proper randomization, ethical research sponsorship, etc.), and in strong adherence with for development of sophisticated personalized diets considering dietary modifications due to microbiota issues, multiple dietary factors (like food, environmental *toxins*) affecting on GI motility and individual health as a whole; toxicity studies for constipation and detoxification effect of NCD in particular. Afterwards, after relevant legislation, registration and control procedures, NCD-based products might be implemented as novel treatments and prebiotic dietary supplement ensuring the quality and truthful marketing.

### Consolidation of the PPPM concept

The results of the study are potentially applicable for creating products—food supplements, develop safe and effective person-related treatments for constipation beneficial for individual outcomes with particular application in elderly, and on the other hand, be part of large concept within *interactome* in order to suggest a healthy lifestyle and sustainable well-being and aging, and also to be a contribution to the understanding of issues of irritable bowel, inflammatory bowel disease (IBD) and visceral pain cancerogenesis.

### Preventive medical approach

Translation of the obtained data on animal model to human organism may allow to consider diet correction with nanoceria additives in particular in elderly against constipation and related bowel diseases.

The results have a potential for preventions a wide scope of bowel diseases and CRC and for promotion of health in the integrated vision of interactome.

### Personalized medical approach

Designing person-related smart physiologic low-dose treatments as important impacts to personalized dietology and is a challenge for medicine of future. Clinical study is necessary for stratifications of potential responders to formulate clear personalized application. Considering biosafety of ceria nanoparticles, the group of potential patients (consumers) can be large.

### Predictive medical approach

Developing the panel of bowel diseases assessment biomarkers from the point of view of extensive vision including gut-brain axis (GBA), intestine microbiota, psyche, stress, emotions, pain, physical activity and molecular and cellular mechanisms is an important point. Development and validation of questionnaires for diagnosis of motility disorders as irritable bowel syndrome, relevant animal modeling using appropriate techniques as *in vivo* imaging (ultrasound, endoscopy, etc) and methods of translating obtained data to human organism should initiate related research towards study of CNS and the ENS within the wall of the gastrointestinal tract and microbiome that can give deeper insight for cancer microenvironment and be a source of potential biomarkers.

## Expert recommendations

With the concluding points, we can formulate the following proposals (expert recommendations):

- For the European Union (EU): create an international research project to study the biomedical effects of nanoceria offer the prospect of its use as a UV protectant, a drug with antiviral, antibacterial and antifungal activity, as well as means capable of reducing the level of oxidative stress in diverse tissues of human body.
- Initiate discussion to suggest project for preventions a wide scope of bowel diseases and CRC and for promotion of health in the integrated vision of interactome.

## Conclusions

1. The index of motor activity decreases in the stomach and colon in rats with age.
2. The amplitude, the phase of contraction and phase relaxation of the contractions caused by hyperpotassium solution decreased in smooth muscle of the colon in old rats.
3. The administration of nanocrystalline cerium dioxide increased index of motor activity of spontaneous and stimulated motility of the stomach and colon in rats of both age.
4. In old rats, nanocrystalline cerium dioxide evoked a significant increase the amplitude, phase contraction and reduction of contractions of the colon, activated by depolarization of the plasma membrane in old rats.
5. Nanocrystalline cerium dioxide increased the amplitude, phase contraction, phase ratio and tonic components of the acetylcholine contractile responses of smooth muscles of the colon in old rats.
6. Obtained results can be the basis for creating a new laxatives based on nanocrystalline cerium dioxide.

## Abbreviations

PPPM: Predictive, preventive and personalized medicine
NCD: Nanocrystalline cerium dioxide
IMA: Index of motor activity
CNS: Central nervous system
ENS: Enteric nervous system
DRG: Dorsal root ganglia
